# Association between serum ferritin and bone mineral density in US adults

**DOI:** 10.1186/s13018-022-03357-1

**Published:** 2022-11-16

**Authors:** Peng Peng, Fangjun Xiao, Shihua Gao, Weihua Fang, Tianye Lin, Wei He, Qiushi Wei

**Affiliations:** 1 Guangzhou University of Chinese Medicine, Guangzhou, China; 2 Guangdong Research Institute for Orthopedics and Traumatology of Chinese Medicine, NO.261, Longxi Road, Liwan District, Guangzhou, 510378 People’s Republic of China; 3 Department of Orthopaedics, The Third Affiliated Hospital, Guangzhou University of Chinese Medicine, Guangzhou, China

**Keywords:** Serum ferritin, Females, Bone mineral density (BMD), National Health and Nutrition Examination Survey (NHANES), Cross-sectional study

## Abstract

**Background:**

The association between serum ferritin and bone mineral density (BMD) is still controversial. This study aims to investigate the association of serum ferritin level with BMD in US adults.

**Methods:**

We conducted a cross-sectional study consisting of 8445 participants from National Health and Nutrition Examination Survey. Serum ferritin and lumbar spine BMD were used as independent variables and dependent variables, respectively. We evaluated the association between serum ferritin and lumbar spine BMD through a weighted multivariable linear regression model. Subgroup and interaction analysis was also performed in this study.

**Results:**

After adjusting for other confounding factors, serum ferritin was negatively correlated with lumbar spine BMD [*β* =  − 0.090, 95% CI (− 0.135, − 0.045)]. Further subgroup analysis found that the strongest negative association mainly exists in females aged over 45 years [*β* =  − 0.169, 95% CI (− 0.259, − 0.079)], and this association is not significant in other groups.

**Conclusions:**

The results found that the association between serum ferritin and lumber spine BMD differed by gender and age. Increased level of serum ferritin may indicate a higher risk of osteoporosis or osteopenia in females aged over 45 years.

## Introduction

Osteoporosis is a complex and chronic disorder, resulting in a progressive reduction in bone strength and enhanced bone fragility with susceptibility to fractures [1, 2]. Osteoporotic fractures are associated with excess mortality and decreased functional capacity and quality of life [3]. Osteoporotic fractures have a huge impact economically, in addition to their effect on health: The cost to the US economy is around $17.9 billion per annum, with the burden to the UK being almost £4 billion [4]. Low bone mineral density (BMD) is an important risk factor of fracture, and treatment is strongly recommended in those with a BMD below a critical value [5]. Therefore, identifying risk factors of low BMD is vital for the prevention and management of osteoporosis.

Ferritin is a large protein formed by apoferritin and iron core Fe^3+^ [6]. Ferritin plays a key role in the regulation of iron metabolism, and it can reflect iron stores in individuals [7–9]. The World Health Organization (WHO) currently defines iron overload as ferritin concentrations > 200 for males and > 150 for females of all ages above 5 years [10]. Iron overload has been thought to be associated with diseases such as cancers, heart attack, heart failure and diabetes mellitus [11, 12]. Several studies have also shown that the incidence of osteoporosis and fractures increases significantly in diseases related to iron overload, such as hemochromatosis, thalassemia, and cirrhosis [13–15]. In this regard, there is ongoing research involved in detecting of the association of serum ferritin with BMD. Two studies reported that serum ferritin was inversely associated with the BMD values at the lumbar spine and femur neck in Korean women aged over 45 years [16, 17]. However, another study suggested that serum ferritin was positively associated with BMD of the total lumbar spine, total femur, and femur neck in elderly South Korean men [18]. A negative and linear association between serum ferritin and BMD was demonstrated in aged 12 to 49 American women; however, some important factors affecting bone metabolism, including serum alkaline phosphatase (ALP), serum calcium, serum uric acid, were not fully adjusted as confounding factors [19].

Accordingly, the aim of this study was to analyzed the association between serum ferritin levels and lumbar spine BMD in US adults using data from the nationally representative National Health and Nutrition Examination Survey (NHANES) database (1999–2006).

## Materials and methods

### Study population

The National Health and Nutritional Examination Survey (NHANES) is a population-based national survey, providing information regarding the nutrition and health of the American population using a complex, stratified, multistage, clustered probability sampling design. The NHANES database is available publicly at www.cdc.gov/nchs/nhanes. In this study, we collected data in four cycles of NHANES, from 1999 to 2006.

**Fig. 1:**
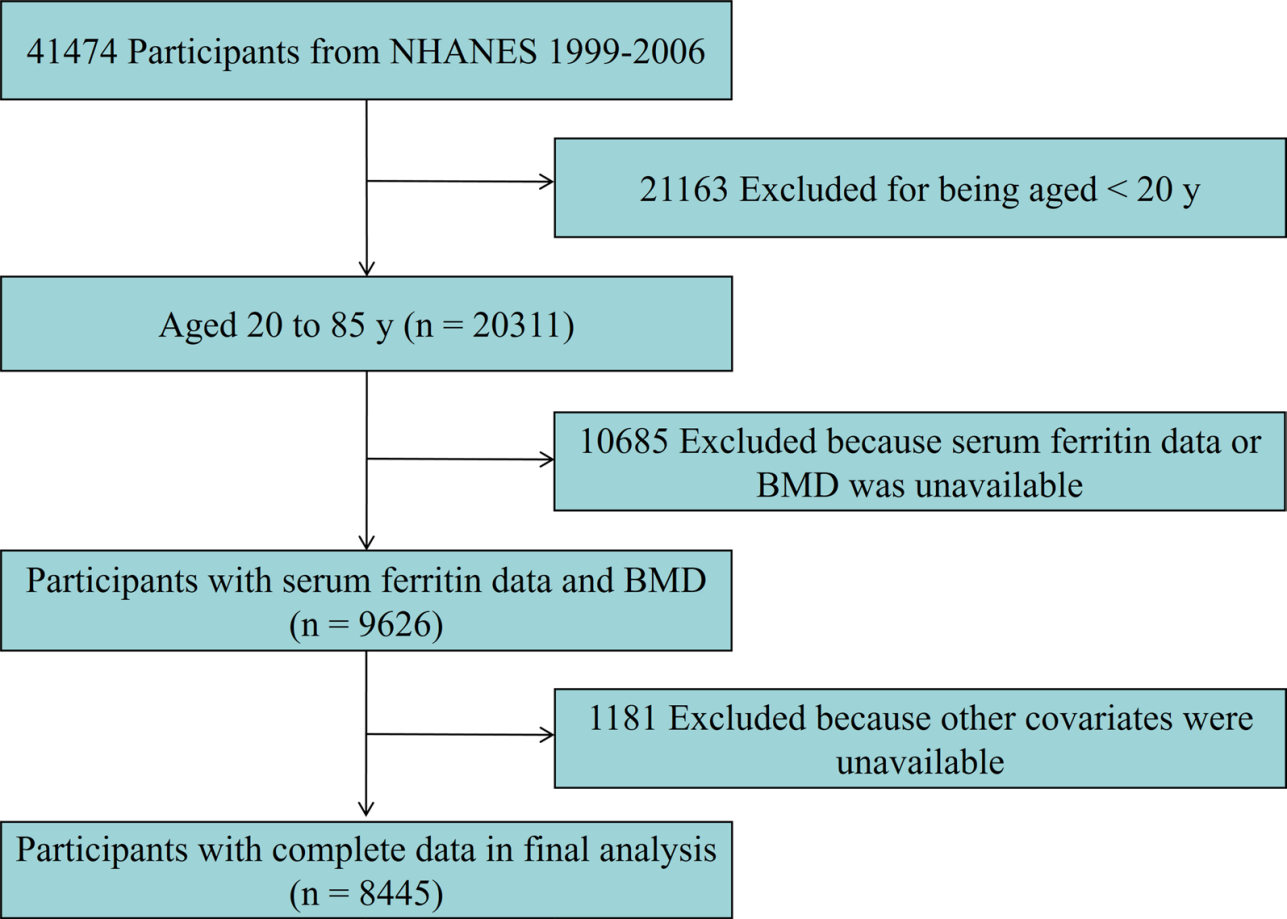
Flowchart of sample selection

In total, 41,474 individuals participated in the health examination surveys between 1999 and 2006. The study population was restricted to adults aged 20–85 years (*n* = 20,311). Of the eligible group, 10,685 individuals were missing data on serum ferritin or BMD, and another 1181 were missing data on other covariate. Finally, 8445 participants were included in the analysis after applying these exclusion criteria (Fig. 1).

### Variables

In this study, the independent variable was serum ferritin. The dependent variable was lumbar spine BMD measured by the dual-energy X-ray (DEXA) scans. We selected these confounders on the basis of their associations with the outcomes of interest or a change in effect estimate of more than 10% [20]. The following variables were included in final analysis as covariates: age, gender, race, education, physical activity, body mass index, serum calcium, total cholesterol, serum albumin, alkaline phosphatase, and serum uric acid. The examination parts related to physiological, clinical, and laboratory evaluations were all carried out by well-trained medical experts. The detailed information of each variable is publicly available at www.cdc.gov/nchs/nhanes.

### Statistical analysis

All analyses used NHANES examination sample weights that adjust for nonresponse, noncoverage, and unequal probabilities of selection. In the descriptive analysis, continuous variables were reported as mean ± standard deviation; categorical variables were reported as percentages. Weighted multivariable linear regression model was used to evaluate the association between serum ferritin and lumbar spine BMD. We constructed three distinct models using weighted univariate and multivariable linear regression models. Interaction and stratified analyses were conducted according to age and gender. *P* values less than 0.05 (two-sided) were considered statistically significant. Modeling was performed with the statistical software packages R (http://www.R-project.org, The R Foundation).

## Results

A total of 8,445 participants aged 20–85 years were included in this study. The weighted socio-demographic and medical characteristics of these participants subclassified based on serum ferritin tertiles (*Q*1: 3–44 ng/mL; *Q*2: 45–108 ng/mL; *Q*3: 109–365 ng/mL) are shown in Table 1. Participants in the highest serum ferritin tertiles were more likely to be men, whites, with lower values of lumber spine BMD and higher total cholesterol, alkaline phosphatase, and serum uric acid.

**Table 1:** Weighted characteristics of 8445 participants included in this study

Serum ferritin (ng/mL)	Total	*Q*1 (3–44)	*Q*2 (45–108)	*Q*3 (109–365)
*N*, unweighted	8445	2806	2799	2840
Age (years)	42.54 ± 15.33	39.15 ± 13.76	42.62 ± 15.78	46.18 ± 15.61
Gender (%)				
Male	34.41	8.59	30.83	66.67
Female	65.59	91.41	69.17	33.33
Race (%)				
White	71.27	68.61	72.92	72.36
Black	10.33	11.41	8.79	10.82
Mexican American	7.50	8.93	6.91	6.60
Other Hispanic	6.43	6.56	7.22	5.42
Other ethnicity	4.48	4.49	4.17	4.81
Education (%)				
Lower than high school	18.97	16.98	18.87	21.25
High school	24.49	23.38	22.63	27.75
More than high school	56.55	59.64	58.51	51.00
Physical activity (%)				
Sedentary	17.79	16.40	18.30	18.77
Low	28.51	29.17	27.74	28.61
Moderate	20.01	21.37	20.11	18.43
High	33.69	33.06	33.85	34.19
Body mass index (kg/m^2^)	28.03 ± 6.68	27.53 ± 6.81	27.89 ± 6.82	28.72 ± 6.32
Serum calcium (mg/dl)	9.44 ± 0.38	9.37 ± 0.37	9.47 ± 0.37	9.50 ± 0.39
Total cholesterol (mg/dl)	197.29 ± 39.95	191.24 ± 37.33	197.73 ± 38.90	203.44 ± 42.79
Serum albumin (g/dl)	4.34 ± 0.33	4.26 ± 0.31	4.35 ± 0.33	4.23 ± 0.33
Alkaline phosphatase (U/L)	70.39 ± 25.12	65.76 ± 22.56	70.64 ± 24.42	75.20 ± 27.50
Serum uric acid (mg/dl)	5.10 ± 1.39	4.53 ± 1.20	5.04 ± 1.29	5.80 ± 1.37
Lumbar spine BMD (mg/cm^2^)	1052.61 ± 148.65	1065.40 ± 138.81	1051.22 ± 147.40	1040.10 ± 158.95

Mean ± SD for continuous variables and % for categorical variables

Weighted multivariable linear regression model was constructed (Table 2). In the unadjusted model, serum ferritin was negatively correlated with lumbar spine BMD [*β* =  − 0.107, 95% CI (− 0.148, − 0.067)]. After adjusting for confounding factors, this negative association still existed in Model 2 [*β* =  − 0.087, 95% CI (− 0.133, − 0.041)] and Model 3 [*β* =  − 0.090, 95% CI (− 0.135, − 0.045)]. Stratified by tertiles of serum ferritin, the trend test remained significant between them (*P* for trend < 0.0001).

**Table 2:** Association of serum ferritin with lumbar spine bone mineral density in 8445 participants aged 20–85 years

	Model 1 *β* (95% CI) *P* value	Model 2 *β* (95% CI) *P* value	Model 3 *β* (95% CI) *P* value
Serum ferritin (ng/mL)	− 0.107 (− 0.148, − 0.067)***	− 0.087 (− 0.133, − 0.041)***	− 0.090 (− 0.135, − 0.045)***
*Q*1	Reference	Reference	Reference
*Q*2	− 14.187 (− 21.819, − 6.556)**	− 10.094 (− 17.675, − 2.513)**	− 7.979 (− 15.363, − 0.595)*
*Q*3	− 25.308 (− 33.129, − 17.488)***	− 22.006 (− 30.836, − 13.177)***	− 20.994 (− 29.720, − 12.269)***
*P* for trend	< 0.0001	< 0.0001	< 0.0001

Model 1: no covariates were adjusted. Model 2: age, gender, and race were adjusted. Model 3: age, gender, race, education, physical activity, body mass index, serum calcium, total cholesterol, serum albumin, alkaline phosphatase, or serum uric acid were adjusted

*P < 0.05, **P < 0.01, ***P < 0.001

On subgroup analysis (Table 3), we observed the association between serum ferritin and lumbar spine BMD stratified by demographic variables. When stratified by gender, a significant negative association existed in females [*β* =  − 0.089, 95% CI (− 0.155, − 0.024)], not in males [*β* =  − 0.057, 95% CI (− 0.120, − 0.006)]. When stratified by age, serum ferritin was negatively correlated with lumbar spine BMD in participants aged over 45 years [*β* =  − 0.097, 95% CI (− 0.159, − 0.036)], and no significant association was found in participants aged below 45 years [*β* =  − 0.062, 95% CI (− 0.128, 0.004)]. We further observed that the association between serum ferritin levels and lumbar spine BMD mainly exists in females aged over 45 years [*β* =  − 0.169, 95% CI (− 0.259, − 0.079)]. This negative relationship was not significant in other subgroups.

**Table 3:** Subgroup analysis of serum ferritin with lumbar spine bone mineral density, stratified by age and gender

Subgroup analysis	*β* (95% CI) *P* value	*P* for interaction
Age, years		0.0249
Age < 45	− 0.062 (− 0.128, 0.004)	
Age ≧ 45	− 0.097 (− 0.159, − 0.036)**	
Gender		0.0007
Male	− 0.057 (− 0.120, − 0.006)	
Female	− 0.089 (− 0.155, − 0.024)**	
Age * gender		0.0461
Female ≧ 45	− 0.169 (− 0.259, − 0.079)**	
Female < 45	− 0.080 (− 0.177, 0.016)	
Male ≧ 45	− 0.021 (− 0.105, 0.064)	
Male < 45	− 0.062 (− 0.154, 0.031)	

Each stratification adjusted for all the factors (age, gender, race, education, physical activity, serum calcium, total cholesterol, serum albumin, alkaline phosphatase, or serum uric acid) except the stratification factor itself

**P < 0.01

Interaction analyses revealed that the association between serum ferritin levels and lumbar spine BMD was modified by age and gender (Table 3). The association between serum ferritin and lumbar spine BMD was stronger among participants aged over 45 years (*β* =  − 0.097 vs. − 0.062, *P*_int_ = 0.0249), among female (*β* =  − 0.089 vs. − 0.057, *P*_int_ = 0.0007). Also, the female aged over 45 years had the highest estimate value between serum ferritin and lumbar spine BMD than other groups (*β* =  − 0.169).

## Discussion

This study was set out to investigate whether serum ferritin is independently associated with lumbar spine BMD. The studied population was a nationally representative and large sample of US adults aged 20–85 years. We found that serum ferritin concentration was negatively associated with lumbar spine BMD in US adults, particularly in females aged over 45 years.

Ferritin is an iron storage protein that regulated post-transcriptionally by cellular iron status via iron responsive elements in its messenger RNA [21]. Serum ferritin level is a routinely available indicator with well-described associations with iron status, and it has been widely used as an indicator of iron overload [22, 23]. Osteoporosis is a metabolic disease characterized by a systemic impairment of bone mass and results from the imbalance between bone resorption and bone formation [24]. Some biological studies have been conducted to confirm the exact effects of iron overload on osteoporosis. Yuan et al. [25] showed that iron accumulation inhibited mesenchymal stem cells (MSCs) quantity and decreased bone mineral density and spatial structural parameters in vivo mice model. He et al. [26] noted that iron overload probably inhibited osteoblast function through higher oxidative stress following increased intracellular iron concentration. On the other hand, various studies reported that iron overload stimulated osteoclast differentiation [27, 28] and aggravated the effects of ovariectomy on bone mass [29]. Meanwhile, some clinical studies have been conducted in order to determine the association between osteoporosis and iron overload-related diseases. In subjects with genetic hemochromatosis, the femur neck BMD appears to fall with rising hepatic iron concentration, and osteoporosis was highly influenced by the degree of iron overload which plays an independent role in the acceleration of bone loss [15, 30]. Iron overload is one of the most important factors in the development of thalassemia-associated osteoporosis and exerts both direct and indirect effects on bone metabolism [31].

Thus, a number of studies focus on the association of serum ferritin with BMD. The negative relationship between serum ferritin and BMD was demonstrated in Korean women [16, 17]; nevertheless, a positive relationship was found in elderly Korean men [18]. In a population-based cross-sectional study, an inverse association of serum ferritin with BMD was detected in American women aged 12–49 years [19]. However, some important factors affecting bone metabolism, including serum alkaline phosphatase (ALP), serum calcium, serum uric acid, were not fully adjusted as confounding factors evidence. In this study, we controlled for the potential confounding variables including demographic, nutrition status, and bone metabolism. In agreement with previous studies [16, 17], our findings indicated that a higher serum ferritin level was associated with a lower lumbar spine BMD in females aged over 45 years. On the other hand, a positive association between ferritin and BMD was found in elderly in South Korean and Iran [18, 32]. We speculated that the correlation between ferritin and BMD may be affected by different populations. Therefore, more prospective clinical studies are needed to confirm the association between serum ferritin and lumbar spine BMD.

Age-related changes of BMD are demonstrated to be influenced by gender, hormonal change, and ethnicity. Specifically, age-related decline in BMD is more pronounced in women than in men [33]. In addition, several studies showed a significant bone loss even in perimenopausal women prior to their menopause [34, 35]. In this regard, we adopted 45 years old as the cutoff points for age stratification both in women and men to obtain a comparable analysis in this study. Similar to previous studies [16, 17], a stronger negative association between serum ferritin and lumbar spine BMD was observed only in women aged over 45 years. The discrepancy could be explained by sudden loss of estrogen in these women, besides gender difference. Decreased estrogen level led to a corresponding decrease in the function of iron response elements, followed by increased iron storage in the body [14, 36]. In addition, estrogen deficiency during menopause can lead to bone loss through complex interplay of hormones and cytokines that converge to disrupt the process of bone remodeling [37]. Therefore, these present results further confirmed the positive association between iron overload and BMD.

In this study, we used the NHANES database, which includes a representative sample of multiethnic population. The large sample size allows us to better conduct subgroup analysis. In addition, we selected the population by rigorous inclusion criteria and adjusted for potential confounding factors as much as possible. However, it is important to acknowledge the limitations of our study. Firstly, the cross-sectional design of our study means that it impossible to determine the causal relationship between serum ferritin and lumbar spine BMD. Secondly, the lumbar spine, the intertrochanteric area, and the femoral neck are currently the most commonly used regions for evaluation of BMD. However, BMD of the intertrochanteric area and the femoral neck were not available in most of the included participants and we only examined the association between serum ferritin and lumbar spine BMD. Thirdly, although we adjusted for potential known confounding factors, residual or unmeasured confounder may alter our observed results. Therefore, further prospective clinical and basic mechanistic studies are needed to confirm the association between serum ferritin and lumbar spine BMD.

In conclusion, this study demonstrated that serum ferritin level was negatively correlated with lumbar spine BMD in females aged over 45 years. Increased level of serum ferritin may indicate a higher risk of osteoporosis or osteopenia in these women. Further basic and clinical studies are needed to clarify the exact effect of serum ferritin on lumbar spine BMD.

## Abbreviations

BMD: Bone mineral density
NHANES: National Health and Nutrition Examination Survey
